# Conspiracy beliefs and COVID-19 guideline adherence in adolescent psychiatric outpatients: the predictive role of adverse childhood experiences

**DOI:** 10.1186/s13034-022-00554-y

**Published:** 2023-01-24

**Authors:** Andreas Goreis, Bettina Pfeffer, Heidi Elisabeth Zesch, Diana Klinger, Tamara Reiner, Mercedes M. Bock, Susanne Ohmann, Petra Sackl-Pammer, Sonja Werneck-Rohrer, Harald Eder, Katrin Skala, Klara Czernin, Dunja Mairhofer, Bernhard Rohringer, Carolin Bedus, Ronja Lipp, Christine Vesely, Paul L. Plener, Oswald D. Kothgassner

**Affiliations:** 1 Department of Child and Adolescent Psychiatry, Medical University of Vienna, Vienna, Austria; 2 Comprehensive Center for Pediatrics (CCP), Medical University of Vienna, Vienna, Austria; 3 Psychosocial Services Vienna, Vienna, Austria; 4 Department of Child- and Adolescent Psychiatry and Psychotherapy, Medical University of Ulm, Ulm, Germany

**Keywords:** Conspiracy beliefs, COVID-19, Adolescents, Mental health, Guideline adherence, Childhood Trauma, Adverse childhood experiences

## Abstract

**Background:**

Conspiracy beliefs have become widespread throughout the COVID-19 pandemic. Previous studies have shown that endorsing conspiracy beliefs leads to lower protective guideline adherence (i.e., wearing face masks), posing a threat to public health measures. The current study expands this research across the lifespan, i.e., in a sample of adolescents with mental health problems. Here, we investigated the association between conspiracy beliefs and guideline adherence while also exploring the predictors of conspiracy beliefs.

**Methods:**

*N* = 93 adolescent psychiatric outpatients (57% female, mean age: 15.8) were assessed using anonymous paper–pencil questionnaires. Endorsement of generic and COVID-19 conspiracy beliefs was assessed, in addition to items measuring adherence to protective guidelines and mental health (stress, depressive symptoms, emotional/behavioral problems, and adverse childhood experiences). Multiple regressions and supervised machine learning (conditional random forests) were used for analyses.

**Results:**

Fourteen percent of our sample fully endorsed at least one COVID-19 conspiracy theory, while protective guidelines adherence was relatively high (*M* = 4.92, on a scale from 1 to 7). The endorsement of COVID-19 conspiracy beliefs—but not of generic conspiracy beliefs—was associated with lower guideline adherence (β = − 0.32, 95% CI − 0.53 to − 0.11, *p* < .001). Conditional random forests suggested that adverse childhood experiences and peer and conduct problems were relevant predictors of both conspiracy belief categories.

**Conclusion:**

While a significant proportion of our sample of adolescents in psychiatric treatment endorsed conspiracy beliefs, the majority did not. Furthermore, and to some degree, contrary to public perception, we found that adolescents show relatively good adherence to public health measures—even while experiencing a high degree of mental distress. The predictive value of adverse childhood experiences and peer/conduct problems for conspiracy beliefs might be explained by compensatory mechanisms to ensure the safety, structure, and inclusion that conspiracies provide.

**Supplementary Information:**

The online version contains supplementary material available at 10.1186/s13034-022-00554-y.

## Background

As of 2022, the COVID-19 pandemic represents an ongoing threat to public health. After the WHO declared this disease a pandemic in March 2020, several government-mandated lockdowns/shutdowns with a severe impact on everyday life were implemented as a measure to prevent infection [1]. Although such measures were necessary to prevent an even more significant escalation of the public health care emergency, the disruption of routine life, confinement, and prolonged social isolation has been shown to negatively affect mental health and well-being in the general population (for a review, see [2]). A plethora of studies also showed that children and adolescents—who are more at risk of developing mental health problems [3]—are particularly affected by the adverse effects of confinement [4–9]. For example, a recent study conducted in Austria [7] reported that in a sample of 1,500 adolescents, 58% reported clinically relevant depressive symptoms, and 46% reported anxiety symptoms one and a half years into the pandemic.

Epidemiological studies from before the pandemic already indicated increasingly high levels of mental health problems in young people in Austria (e.g., [10]). Yet, one study one year into the pandemic found that young people—compared to before the pandemic—had increases in clinically relevant depressive and anxiety symptoms by 4.5–5 and 1.8–threefold likelihoods, respectively [11, 12]. Moreover, as was the case before the pandemic, gender differences had an impact on mental health during the pandemic as well. Indeed, a recent review concluded that mental health symptoms increased more in women, particularly in young women [13].

Concurrently with the spread of the virus, fake news, as well as conspiracy beliefs about the origin or alleged motivation behind the pandemic, became a viral phenomenon [14]. Conspiracy beliefs are, in the broadest sense, the attempt to explain a situation, an event, or a development through a conspiracy, i.e., through the purposeful, conspiratorial activity of a mostly small group of actors for an often illegal or illegitimate purpose [15, 16]. Conspiracy beliefs may further be divided into specific and generic conspiracy beliefs, with generic referring to beliefs about a widespread conspiracy theory behind events (e.g., “Certain significant events have been the result of the activity of a small group who secretly manipulate world events.”, Brotherton et al. [17], p. 5). In contrast, specific conspiracy beliefs refer to beliefs about specific real-world events and situations, e.g., staging of the moon landing or in the current context: regarding the origin of COVID-19 (for a review regarding different types of conspiracy beliefs, see Goreis & Voracek, [18]). For example, a recent study found that in an adult sample in German-speaking countries, 10% fully endorsed, and another 20% somewhat endorsed conspiracy beliefs about COVID-19 [19].

Notably, the endorsement of conspiracy beliefs has been connected to worse adherence or even the refusal to adhere to government-mandated protective measures (e.g., social distancing, wearing protective face masks) during the pandemic [20, 21]. As government-mandated measures are effective [22], higher non-adherence rates have been found to burden health care systems indirectly and additionally (e.g., through higher rates of new infections). In addition, non-adherence is also negatively associated with economic recovery from the pandemic (see [23], for a comprehensive report). Conspiracy beliefs—both generic and COVID-19 specific—may, therefore, indirectly pose an additional threat to public health when a proportion of the population endorses them, undermining government-mandated actions against the pandemic. Indeed, studies have confirmed the negative association between conspiracy beliefs and adult adherence to protective measures [24–27]. What is unknown, however, is what young people think about conspiracy theories and how these beliefs may affect their adherence to protective measures against COVID-19. With the notable exception of Jolley et al. [28], research on conspiracy beliefs across the lifespan is nonexistent, especially in young people who are already facing adversity: those in psychiatric care. Therefore, the present study aimed to expand this area of research by investigating generic and COVID-19 specific conspiracy beliefs, their effects on protective adherence, and mental health predictors of conspiracy beliefs in a sample of German-speaking adolescent psychiatric outpatients.

### Conspiracy beliefs and mental health

Several factors, such as personality traits, social media use, and trust in authorities, have been suggested to drive the endorsement of conspiracy beliefs during the current pandemic (for reviews, see [29, 30]). However, the association between conspiracy beliefs and mental health (i.e., the predictive value of mental health in this context) is not yet fully understood. Depressive symptoms [14, 31] and stress [32, 33], for example, were associated with greater endorsement of conspiracy beliefs during the pandemic. Before the pandemic, research on mental health and its connection to conspiracy beliefs was relatively sparse: one report found that perceived stress and trait anxiety had small effects on beliefs [34], while others focused on the predictive value of clinical or maladaptive personality traits, such as schizotypy and negative affectivity (e.g., [35]). Some argue that conspiracies take the function of coping mechanisms in uncertain, uncontrollable, and traumatic times—which can elicit stress—and that conspiracy beliefs may help people who need to make sense of an uncertain world [16, 18]. Following this line of thought, it could be assumed that people who need effective coping, i.e., due to mental health disorders, should tend to show an increased prevalence of conspiracy beliefs.

Less is known about conduct or peer problems and their association with conspiracy beliefs. However, in adult samples, there are findings of positive links between conspiracy beliefs and related symptoms: defiant/rule-breaking behavior [36] and antisocial and impulsive behavior [37]. Another study has shown a causal link between ostracism—often reported by adolescents with peer problems and traumatic experiences—and endorsement of conspiracies in a general population sample [38]. Adults with diagnosed mental disorders also reported higher endorsement than those without [39]. As outlined, the majority of investigations of predictors of conspiracy beliefs in this field have been conducted in adults.

### Current study

The goal of the current study was to focus on individuals who were primarily assessed with a focus on their mental health impairment and deterioration of well-being caused by the measures against the COVID-19 pandemic: young people, especially those who require treatment because of mental health problems. To date, no study has assessed the opinions of young people about viral conspiracy beliefs pertaining to the pandemic. Additionally, no investigations in this matter exist in the context of a population particularly at risk: children and adolescents in psychiatric care. We, therefore, assessed conspiracy beliefs (generic ones and those on COVID-19), adherence to protective guidelines, and mental health indicators (depressive symptoms, stress, behavioral and conduct problems, as well as adverse childhood experiences) in a sample of adolescents currently in treatment at a psychiatric outpatient clinic. Our study was conducted during the first half of 2022 (around two years into the pandemic) when no lockdowns/shutdowns were in place, and schools were open in Austria.

First, we aimed to depict the prevalence of conspiracy beliefs reported by adolescents with mental health problems. Endorsement rates of conspiracies are particularly relevant as some have argued that conspiracy beliefs may form as a repercussion of pathology (e.g., [40]). If this was the case, the prevalence of conspiracy beliefs in adolescents with mental health problems should be higher than that other studies have reported in general population samples (e.g., in [19]). The current adherence to protective measures is also a relevant factor in our study—do adolescents with mental health problems generally report a low adherence to such measures, as was reported before [36, 41]? Furthermore, we hypothesized that—as is the case in adult samples and a barrier to successful pandemic management [24–27, 42]—a higher endorsement of generic or COVID-19 conspiracy beliefs will be associated with lower adherence to protective measures. We also aimed to explore the predictive value of mental health indicators on conspiracy beliefs. In summary, our aim was to investigate whether and which mental health variables would predict conspiracy beliefs in adolescents with mental health problems requiring treatment. Even though factors such as stress and depressive symptoms were previously associated with conspiracy beliefs in adults [29], our exploratory analyses of mental health predictors are not based on a robust theoretical background. Due to the nature of our sample (i.e., they are being investigated because they experienced mental health problems), we expected relatively high values in adverse mental health indicators. Nonetheless, explorations of mental health predictors could potentially identify factors relevant for follow-up investigations or interventions.

## Methods

### Participants and procedure

A total of 93 German-speaking adolescents (age range: 11–18) participated in the current study, which was conducted between January and July 2022. All of them were currently in outpatient care at the Department of Child and Adolescent Psychiatry at the Medical University of Vienna. The department and its outpatient clinic are part of the Viennese public hospital infrastructure. It is mandated to provide services for all children/adolescents within the Vienna residential area—without any restrictions regarding disorders or conditions. We used a consecutive sampling strategy whereby all patients were approached while waiting for or during their appointment at the outpatient clinic. There was no minimum contact duration to be eligible for inclusion. If patients agreed to participate, questionnaires were handed to them by their psychologists or physicians in charge of treatment. No further inclusion criteria aside from a sufficient command of the German language had to be met. The questionnaires were filled out in the waiting area of the outpatient clinic or at home and returned at the next visit. Participants also received an empty envelope and were instructed to insert the questionnaires after completion, seal the envelope, and place it in a mailbox at the administration desk. Participation was anonymous, and all participants read and provided informed consent before participating. The study was approved by the ethics committee of the Medical University of Vienna (reference number: 2289/2020).

### Instruments

#### Generic conspiracy beliefs

This study used the German version of the 15-item Generic Conspiracy Beliefs Scale (GCBS, 17). The GCBS presents statements about conspiracy theories in a generic and decontextualized way and provides a measure of conspiratorial beliefs that focuses less on specific beliefs (e.g., “Secret organizations communicate with extraterrestrials, but keep this fact from the public”). Participants respond via a 5-point Likert-scale (1 = definitely not true; 5 = definitely true). The English version of the scale was previously validated by Swami et al. [43]; for the German validation, see [44]. Cronbach’s Alpha was 0.94 in the current study.

#### COVID-19 conspiracy beliefs

We used 16 items (in German) that referred to COVID-19 specific conspiracy beliefs, most of which were previously used in Pfeffer et al. [33]. These items were adapted from popular and circulating conspiracy theories such as the involvement of pharmaceutical companies, Bill Gates, foreign powers, or electromagnetic waves (i.e., 5G masts) in the development/spread of the virus. The items were rated on a visual analog scale ranging from 0 (definitely not true) to 100 (definitely true). Similar to Kuhn et al. [19], we additionally collapsed responses along the 0–100 scales where we defined the range of 1–25 to an item as “agree a little”, the range of 26–50 as “agree moderately”, the range of 51–75 as “agree a lot”, and the range of 76–100 as “agree completely”. This categorization of the visual analog scale allows us to fairly compare our endorsement rates to other studies—especially those of [19]. The metric response variables (ranging from 0 to 100) of the COVID-19 beliefs scale were, however, retained for all other analyses. Cronbach’s Alpha of this scale was 0.91.

#### Adherence to protective guidelines

Adherence to mandated government protective guidelines was assessed with five items adapted from Swami & Barron [45]. The items were: (1) Only going outside for food, health reasons, or work/school (only if you cannot work from home/no home-schooling is available), (2) Staying 2 m away from other people when you have to go out, (3) Washing your hands regularly, (4) Not meeting other people, including family and friends you do not share a home with, and (5) Wearing a mask when required. All items were presented in German and rated on a 7-point Likert-scale ranging from 1 (did not adhere to) to 7 (completely adhered to). Following Swami & Barron [45], we computed total scores by taking the mean of the five items. Cronbach’s Alpha was 0.72 in the current study’s German versions of the items.

#### Depressive Symptoms

We used the Beck Depression Inventory II (BDI II) in a German translation [46] to assess depressive symptoms in the last two weeks using 21 individual questions. We chose to use the BDI II in our sample as there is extensive literature on its accuracy and reliability in measuring depressive symptoms in children and adolescents [47]. For each question, four statements with a Likert-scale ranging from 0 to 3 were available for selection, where 3 corresponds to a strong symptom expression. An example item would be: “Pessimism: 0 = I am not despondent about the future, 1 = I am more despondent about the future than usual, 2 = I am despondent and do not expect my situation to get better, 3 = I believe my future is hopeless and will only get worse”. In accordance with the manual, sum scores were computed, and Cronbach’s Alpha was 0.94.

#### Perceived stress

We used the German translation [48] of the Perceived Stress Scale (PSS-10) by Cohen & Williamson [49] to measure perceived stress in the previous month (e.g., “In the last month, how often have you felt that you were unable to control the important things in your life?”). All items were rated on 5-point scale (0 = never; 4 = very often). Cronbach’s Alpha was 0.87.

#### Emotional and behavioral problems

We used the German version of the Strengths and Difficulties Questionnaire (SDQ, [50]) to measure several important domains of adolescent psychopathology. The SDQ has 25 items (e.g., “I try to be nice to other people, their feelings are important to me” and “I am restless, I cannot stay still for long)” that are allocated to five subscales: emotional symptoms, conduct problems, hyperactivity/inattention, peer problems, and prosocial behavior. All items were rated on a 3-point Likert-scale (0 = not true; 2 = certainly true). Higher scores indicate more serious problems—except for prosocial behavior, where higher scores indicate more positive behavior. A total difficulties score can also be obtained by summing up the scores on the four difficulties scales (i.e., all scales but prosocial behaviors). Cronbach’s Alpha was 0.65 for the total score.

#### Adverse childhood experiences

We used the German version of the Childhood Trauma Questionnaire (CTQ, [51]) to assess adverse childhood experiences. The CTQ measures maltreatment in childhood and adolescence, with 25 items (and three validity items) rated on a 5-point scale ranging from 1 = never true to 5 = very often true. The items of its five subscales (i.e., emotional and physical neglect; emotional, physical, and sexual abuse) may be summed up, with a potential range for each subscale from 5 to 25. A total score of all items may also be computed. Cronbach’s Alpha was 0.93 for the CTQ total score.

### Statistical analyses

All analyses were conducted in R 4.0.2 [52]. First, our sample’s demographic characteristics, endorsement rates for generic and COVID-19 specific conspiracy beliefs, adherence to protective guidelines, and mental health variables were computed descriptively. Then, for our confirmatory hypotheses (the association between COVID-19/generic conspiracy beliefs and adherence to guidelines), we ran two multiple regression analyses for each conspiracy beliefs score while including the additional predictors of age and gender in the models. Power analysis using G*Power [53] revealed that at least 90 participants were necessary to find a small effect of *f*^2^ = 0.07 with 80% power in multiple regression analyses with three predictors (option: fixed model, simple regression coefficient) based on an alpha level of 0.05. Model assumptions of the two regression analyses were all met and reported in the Additional file 1:1 and Figures S1 and S2.

We used conditional random forests for our exploratory analyses (the predictive value of mental health on generic and COVID-19 conspiracy beliefs). Conditional random forests are a supervised machine learning technique helpful for exploring associations without a priori predictors or hypotheses. Such models have previously been used in the context of predictors of COVID-19 conspiracies in Braud et al. [32]. They work by repeated resampling from a training dataset, thereby deriving signals (i.e., predictive importance of predictors on the outcome). Multiple decision trees are formed, the algorithm counts if a predictor is relevant or not, and the result will be a list of predictors sorted by importance. This approach is less prone to overfitting, deals well with multicollinearity, and works in small samples [54, 55]. For our exploratory analyses, we thus ran two conditional random forests, one with generic conspiracy beliefs and one with COVID-19 conspiracy beliefs as outcomes—depressive symptoms, perceived stress, emotional/behavioral problems (subscales of the SDQ), and adverse childhood experiences (subscales of the CTQ), age, and gender were defined as predictors. The models were replicated 100 times, and the code for these analyses was adapted from Braud et al. [32]. In the multiple regression analyses, all predictors were scaled (i.e., *z*-transformed). The scores of the GCBS, the COVID-19 specific conspiracy items, and all CTQ subscales were log-transformed before the conditional random forest computations. All data are publicly available without identifying personal data, i.e., without age, gender, education level, and diagnosis on a repository of the Open Science Framework (https://osf.io/4f7w8/, doi:10.17605/OSF.IO/4F7W8).

## Results

### Sample characteristics

In total, *N* = 93 adolescents currently in outpatient care participated in the present study. The majority had a diagnosis in the ICD-10 F4x disorders (44%), followed by F3x (32%) and F6x (17%). See Table 1 for a complete depiction of sociodemographic characteristics and diagnoses.

**Table 1:** Demographic characteristics of the sample (*N* = 93)

Age (*M* (*SD*)), Range	15.80 (1.45), 11–18
Gender *N* (%)	
Female	51 (55%)
Male	26 (28%)
Diverse	7 (8%)
Prefer Not to Say	5 (5%)
Highest Level of Education N (%)	
Primary School	27 (29%)
Secondary School	47 (51%)
Upper Secondary School	5 (5%)
Vocational Job Training	14 (15%)
Relationship Status	
Single	67 (72%)
In a Relationship	20 (22%)
Married	3 (3%)
ICD-10 Disorders Diagnoses (*N* (%))	
F3x Mood [affective] disorders	30 (32%)
F4x Neurotic, Stress-Related and Somatoform Disorders	41 (44%)
F5x Behavioural Syndromes Associated with Physiological Disturbances and Physical Factors	1 (1%)
F6x Disorders of Personality and Behaviour	16 (17%)
F8x Pervasive and Specific Developmental Disorders	3 (3%)
F9x Behavioural and Emotional Disorders	2 (2%)

Age had 6 missing values, Gender 4 missing values, and relationship status 3 missing values. Percentages may not total 100 due to rounding

### Endorsement of conspiracy beliefs

Our sample reported a relatively low endorsement of generic conspiracy beliefs (GCBS scores) with a mean of 1.86 (*SD* = 0.84; potential range: 1–5). Regarding COVID-19 specific beliefs, 14% of all participants did not endorse any conspiracy beliefs at all. However, 14% of the sample also fully endorsed at least one specific conspiracy theory about COVID-19. The specific theories that were endorsed the most were about COVID-19 being exploited by the pharmaceutical industry to enrich itself financially (19% agreed completely), COVID-19 not being worse than the common flu (16% agreed completely), COVID-19 escaping from a research laboratory (10%), and COVID-19 was unintentionally (8%) and intentionally (5%) released as a bioweapon. Overall, the mean for all COVID-19 conspiracy items was 15.94 (*SD* = 17.34; potential range: 0–100). Generic and specific conspiracy beliefs were highly correlated (*r* = 0.86, *p* < 0.001). See Table 2 for an overview of the mean and proportional endorsement rates for each COVID-19 conspiracy belief item.

**Table 2:** Overview of the endorsement rates to COVID-19 specific conspiracy beliefs

	*M* (*SD*)	% With zero endorsement	% Agree a little	% Agree moderately	% Agree a lot	% Agree completely
The pharmaceutical industry exploits COVID-19 to enrich itself financially through medication and vaccinations	36.93 (34.64)	20	29	21	12	19
COVID-19 is no worse than the common flu	31.74 (33.44)	16	42	18	7	16
COVID-19 escaped from a research laboratory	26.11 (31.26)	34	27	12	16	10
COVID-19 was unintentionally released as a bioweapon	21.09 (29.20)	38	30	16	8	8
COVID-19 was intentionally released as a bioweapon	17.22 (27.11)	49	24	13	9	5
The COVID-19 crisis is used by secret societies to establish an authoritarian world order	15.99 (26.59)	50	24	11	10	4
The COVID-19 crisis is supposed to cover up an upcoming economic crisis	15.46 (26.21)	49	26	14	2	8
The COVID-19 crisis is a pretext to restrict civil liberties permanently	14.30 (26.46)	58	21	8	8	6
The COVID-19 crisis is used by Bill Gates to gain more control over the world	11.20 (23.16)	58	26	7	4	4
The COVID-19 crisis is used by governments to establish a dictatorship	10.03 (21.27)	61	24	10	2	3
Polymerase chain reaction (PCR) tests do not work and are used to push up/increase case numbers systematically	10.01 (22.17)	66	20	9	1	4
The COVID-19 crisis and the related measures are supposed to be used to abolish (hard) cash	8.02 (19.27)	64	24	7	3	2
COVID-19 vaccines are used to implant a microchip	7.70 (20.11)	68	20	9	3	0
COVID-19 infection is prevented by using alcohol and nicotine to protect against COVID-19 infection	7.43 (20.14)	71	19	3	3	3
COVID-19 is, among other ways, spread by 5G transmission masts	7.33 (19.08)	73	16	5	3	2
The COVID-19 infection is caused by wearing a face mask	5.49 (17.30)	77	15	2	3	2

### Adherence to COVID-19 guidelines

The majority of participants indicated that they adhere to protective guidelines pertaining to the ongoing pandemic. However, 25% of all participants indicated that they did not adhere to at least one of the five items. The mean of the 5-item scale was 4.92 (*SD* = 1.09; potential range 1–7). The highest agreement was to the item “wearing a mask when required” (*M* = 6.61, *SD* = 0.97), followed by “washing hands regularly” (*M* = 5.68, *SD* = 1.62), “only going outside for food, health reasons, or work/school” (*M* = 4.58, *SD* = 2.12), “staying 2 m away from people” (*M* = 3.98, *SD* = 1.85), and, lastly, “not meeting other people” (*M* = 3.75, *SD* = 2.05).

### Mental health

The mean for depressive symptoms in the BDI-II was 28.53 (*SD* = 14.64), which, according to the manual, indicates moderate depression (with severe depression starting at 29). The mean BDI-II scores were relatively high compared to community samples of healthy German-speaking people (e.g., a mean of 7.69 in [46]) but comparable to a sample of German-speaking adolescent psychiatric patients in Besier et al. [56] who reported a mean of 24.30. The scores regarding perceived stress—as measured by the PSS-10—were above the midpoint (*M* = 2.58, *SD* = 0.81), indicating moderate levels of perceived stress. Regarding emotional and behavioral problems (the results of the SDQ), the total difficulties score amounted to *M* = 18.61 (*SD* = 5.41), which indicates a high amount and is within the 96th percentile rank in a representative and normative study of German adolescents [50]. Participants’ scores were highest on the subscale emotional problems (*M* = 6.23, *SD* = 2.74), followed by peer problems (*M* = 4.04, *SD* = 2.05), hyperactivity/inattention (*M* = 5.56, *SD* = 2.28), and conduct problems (*M* = 2.78, *SD* = 1.84). Prosocial behavior—which is positively scored, i.e., higher values indicate more prosocial behavior—had a mean of 7.52 (*SD* = 1.95), which is within the range of average scores (i.e., 42nd percentile rank) of German-speaking adolescents in Becker et al. [50].

In the CTQ, 60% of participants reported experiences of emotional neglect, 23% physical neglect, 61% reported having experienced emotional abuse, 20% physical abuse, and 14% reported having experienced sexual abuse (according to cutoffs of the scale manual). In the current study, the CTQ total score was *M* = 46.04 (*SD* = 17.61).

### Confirmatory: associations of conspiracy beliefs and adherence to protective guidelines

Results of our multiple regression analyses confirmed that endorsement of COVID-19 specific conspiracy beliefs (β = − 0.32, 95% CI − 0.53 to − 0.11, *p* < 0.001) was negatively associated with adherence to protective guidelines measure (*R*^2^ = 0.14). Generic conspiracy beliefs, however, were not significantly associated with adherence (β = − 0.21, 95% CI − 0.42– 0.00, *p* = 0.054, *R*^2^ = 0.09)). Age and gender did not affect adherence to protective guidelines in both regressions (all *p*s > 0.138).

### Exploratory: associations of conspiracy beliefs and mental health

**Fig. 1:**
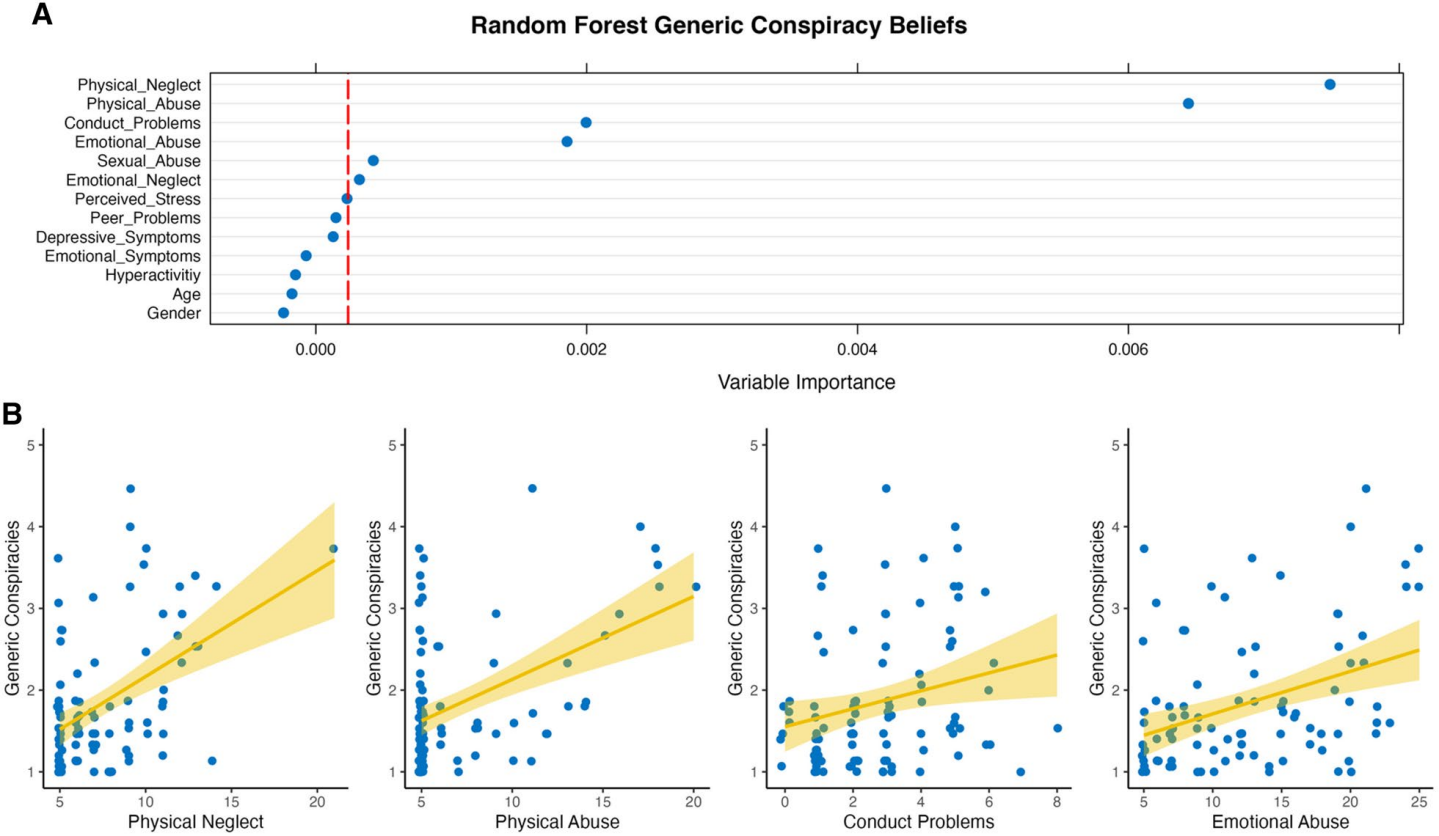
Dot plot of the conditional random forest predicting generic conspiracy beliefs **A** and plotted associations between the five most important predictors of generic conspiracy beliefs **B**

**Fig. 2:**
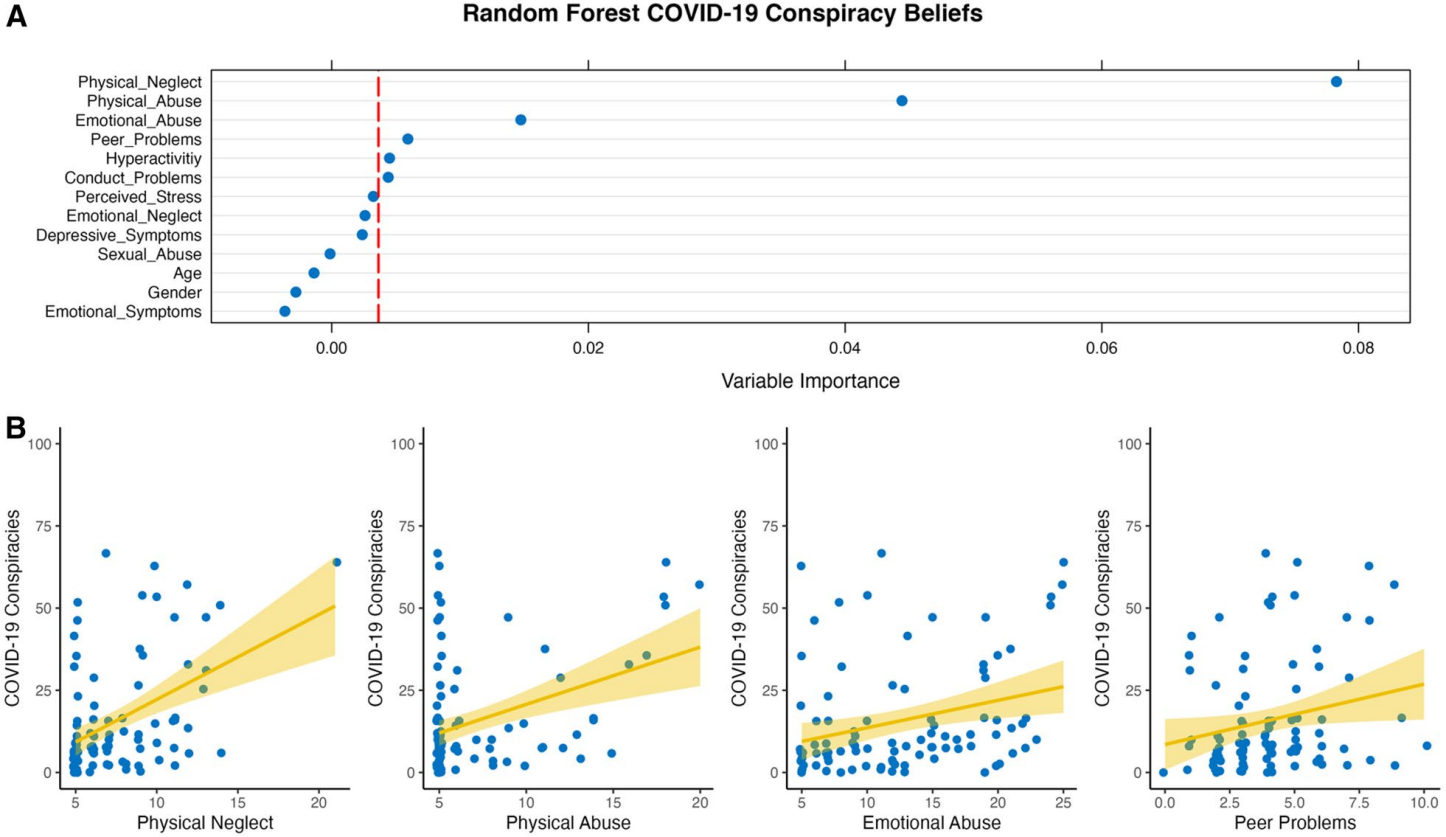
Dot plot of the conditional random forest predicting COVID-19 conspiracy beliefs **A** and plotted associations between the four most important predictors of COVID-19 conspiracy beliefs **B**

The results of the conditional random forest iterations of both conspiracy belief outcomes are depicted in Figs. 1A and 2A. Regarding generic conspiracy beliefs, we found that physical neglect, physical abuse, conduct problems, emotional abuse, sexual abuse, and emotional neglect were relevant predictors above random noise (in that order). RMSE for this model was 0.25 and *R*^2^ = 0.14. For the four most important variables, the association is such that higher scores in physical neglect, physical abuse, conduct problems, and emotional abuse were associated with a higher endorsement of generic conspiracy beliefs (Fig. 2B). Sexual abuse and emotional neglect as a predictor of generic beliefs were dropped from closer inspections due to their relatively low variable importance.

For COVID-19 specific conspiracy beliefs, the results of the conditional random forests are relatively similar—physical neglect, physical abuse, emotional abuse, and peer problems emerged as relevant predictors (in that order, see Fig. 2A). RMSE for this model was 0.24 and *R*^2^ = 0.11. The four variables with the highest predictive power showed that physical neglect/abuse, emotional abuse, and peer problems were all positively associated with endorsement of COVID-19 conspiracy beliefs (Fig. 2B).

## Discussion

The COVID-19 pandemic has confronted societies worldwide with a concurrent spread of conspiracy beliefs. We found that in an adolescent sample that experiences a relatively high amount of mental distress and adversity, the prevalence of generic conspiracy beliefs and COVID-19 specific conspiracy beliefs are relatively low. The score for conspiracy beliefs, i.e., the tendency to endorse conspirational acts by generic/non-specific actors without much context, was on the lower end of the scale (i.e., 1.86 on a scale from 1 to 5). These endorsement rates were lower compared to recent studies of adults using the GCBS to measure generic conspiracy beliefs (e.g., [32, 57]). This difference may be because generic beliefs—even though they are not citing a specific or “real-world” conspiracy—require some form of exposure that was not readily given by our sample due to their young age. This, however, remains speculation as the literature on the topic of age differences is nonexistent. Regarding the COVID-19 specific conspiracies, 14% of our participants fully endorsed at least one COVID-19-related conspiracy belief—a finding very similar to Kuhn et al. [19], where 10% of a representative German-speaking sample reported this quota. In the same paper, the authors also reported the numeric mean of all COVID-19 conspiracy items (ranging from 0 to 100 like in the current study) as 13, whereas it was 16 in our sample. Although not completely comparable due to other item wordings or cultural differences, an English study of adults reported 10–15% of people with consistently high endorsement rates [42]. In summary, our results indicate that most of our sample of adolescents in psychiatric care did not report a very high level of endorsement of any form of conspiracy beliefs. The results were comparable to those using the same or similar instruments in the general population/adult samples. There was, however, a noticeable minority of people who endorsed conspiracy beliefs strongly or at least to some degree.

Regarding the overall adherence to COVID-19 guidelines, our sample was in line with previous work on adolescents [41]: The mean of 4.92 (out of 7) indicated a moderate to a high level of adherence, but not complete adherence. Studies have shown that young adults disagree with protective measures more than old adults [58, 59]. However, our results (and others, e.g., [60]) do not concur with a linear association between age and guideline adherence. Even though adolescents were disproportionately affected by factors such as school closings and mandatory masks in classrooms—and often face mental health issues, such as our sample—they still participate in the public effort to combat the pandemic, potentially more so than young adults (see also [61]).

Our confirmatory analysis regarding the negative association between conspiracy beliefs and adherence to COVID-19 guidelines showed that this was indeed the case in our sample—those who endorsed conspiracies about COVID-19 also reported less adherence (with a medium effect size of β = − 0.32). Of note, this association was found despite our sample’s relatively low endorsement rates of conspiracies. A potential explanation might be that even when individual conspiracies about COVID-19 are not fully endorsed, a level of uncertainty (i.e., residual truth) remains that might affect guideline adherence after all [24]. In addition, generic pre-existing conspiracy beliefs were not significantly associated with adherence in our sample. Generic conspiracy beliefs, however, were strongly associated with our measure of COVID-19 conspiracies, implying a temporal order that could not be tested sufficiently with the design of our study. Endorsement of specific conspiracy beliefs is—necessarily—prone to change with context, whereas some, e.g., Imhoff et al. [62], have argued that generic beliefs are a relatively stable latent disposition. Conspiracy beliefs have circulated even before the COVID-19 pandemic (see [18]), a finding that was probably corroborated by our scores of the generic conspiracy measure. It would be interesting for subsequent research to test, e.g., the effects of experimental manipulations targeting generic beliefs but not specific ones. Overall, our results confirm the significance of conspiracy beliefs and their moderate association with real-world consequences for public health measures and expand this knowledge to adolescents facing mental health issues.

### Conspiracy beliefs and mental health variables

Given the importance of conspiracy beliefs in COVID-19 prevention, we aimed to explore mental health variables that predict conspiracy beliefs in our sample. Descriptively—and by design—we found that our sample reported relatively high levels of mental distress: On average, they experienced moderate depressive symptoms and stress, as well as very high levels of peer problems, hyperactivity, and conduct problems. More than half of the sample reported a history of emotional abuse; in addition to one-fifth who reported physical abuse, 14% reported having experienced sexual abuse. We found that three mental health factors were relevant when predicting generic as well as COVID-19 conspiracy beliefs: adverse childhood experiences (physical neglect, physical abuse, followed by emotional abuse) and conduct, as well as peer problems.

Evidence for the association of adverse childhood experiences and conspiracy endorsement was shown (retrospectively) only in one other study of adults [63], yet with items pertaining to physical and emotional trauma similar to our data. Traumatic events may cause negative alterations in cognition and mood [64]. Therefore, it may be possible that in adolescents with adverse childhood experiences, conspiracy beliefs provide them with a sense of security and control, i.e., a way to fulfill basic psychological needs. A similar explanation could be valid for the predictive power of peer and conduct problems—conspiracy beliefs are a way to socially connect and belong when these needs are not met, as is often the case in people who are ostracized and excluded, i.e., those experiencing peer or conduct problems [38]. Our findings also correspond with another line of research: evidence of the association between anxious attachment styles as a predictor of conspiracy beliefs was reported by Green and Douglas [65]. Notably, none of the other mental health variables recently reported as predictors of conspiracy beliefs in adult samples, such as stress (e.g., 33) and depressive symptoms (e.g., 14), had predictive value in our sample. Even though our participants already experienced high levels of mental distress, conspiracy beliefs were not driven by mental health factors aside from adverse childhood experiences and peer/conduct problems. A significant proportion of the tendency to believe conspiracies may, therefore, be rooted in one’s childhood experiences.

The current study is not without limitations. First, even though our sample comprised a large share of outpatients over the study period (10% or 93 of all 976 outpatients that presented to our clinic from January to July 2022 filled out our questionnaires), we cannot claim that our data is representative. Secondly, responses might have been influenced by social-desirability bias. Even though we attempted to reduce such factors by allowing participants to complete their surveys at home and return them, some participants chose to answer the surveys while waiting for their appointment with supposably limited privacy. Thirdly, our design (and all analyses) was cross-sectional. Even though our confirmatory analyses of the effect of conspiracy beliefs on guidelines adherence were theory-driven and based on previous research, causality cannot and should not be inferred. Our exploratory analyses of the predictors of conspiracy beliefs are similarly limited; however, we want to note that the aim was to identify predictors that may be relevant for future investigations, and no causality was claimed. Finally, as is the case with all COVID-19-related research, the exact time of the study (January–July 2022) and the fluctuating nature of both circulating conspiracy beliefs and the everyday life situations due to the ongoing pandemic (i.e., new virus variants, recent lockdowns/shutdowns, or school-closings) should be considered.

## Conclusions

We provide an overview of the endorsement of conspiracy beliefs and adherence to protective guidelines by a sample previously not queried in this matter: adolescents with mental health problems who presented at a psychiatric outpatient clinic in a German-speaking country. Comparatively, our results showed that conspiracy beliefs are not very widespread in young people. Furthermore, adolescent outpatients in our sample reported adhering to a relatively large degree of protective measures. Higher endorsement of conspiracy beliefs about COVID-19 was linked to lower guideline adherence, implying that—irrespective of age—conspiracy beliefs pose a threat to public health. Therefore, we note that young people should not be neglected in the matter of public health messaging campaigns. Conspiracy beliefs are often disseminated on social media platforms—some of which are predominately used by young people. Reviews of COVID-19 conspiracy beliefs and misinformation and social media [30, 66] previously suggested that health authorities should aim to debunk conspiracy beliefs directly on social media. Furthermore, programs to increase social media users’ media competency to determine reliable information better may be warranted [67], which may also be a feasible aspect to include in the routine treatment of young outpatients.

Our study also adds to the understanding of potential factors associated with conspiracy beliefs in adolescents with mental problems: adverse childhood experiences and peer and conduct problems. Our finding, which suggests that adverse events or interpersonal problems may precede conspiracy endorsement and, subsequently, lower adherence, calls for replication. In their work, clinicians may alleviate relevant mental distress and symptoms and potentially affect how adolescents deal with conspiracies and fake news, thereby having a positive and supportive impact on public health measures.

## Supplementary Information

**Additional file 1. 1: Model assumptions of our confirmatory analyses. Figure S1: Visual check of model assumptions for the multiple regression of COVID-19-specific conspiracy beliefs and adherence to protective guidelines. Figure S2: Visual check of model assumptions for the multiple regression of generic conspiracy beliefs and adherence to protective guidelines.**

## Data Availability

The dataset supporting the conclusions of this article is available in the OSF repository, https://osf.io/4f7w8/.
