# Supplementary material for: Conspiracy beliefs and COVID-19 guideline adherence in adolescent psychiatric outpatients: the predictive role of adverse childhood experiences
Source: Child Adolesc Psychiatry Ment Health. 2023 Jan 24;17:13. doi: 10.1186/s13034-022-00554-y (PMC9873214; doi:10.1186/s13034-022-00554-y)

**Supplementary Material**

**Conspiracy Beliefs and COVID-19 Guideline Adherence in Adolescent Psychiatric Outpatients: The Predictive Role of Adverse Childhood Experiences**

Andreas Goreis^1,2^, Bettina Pfeffer^1,2^, Heidi Zesch^1,2^, Diana Klinger^1,2^, Tamara Reiner^1,2^, Mercedes M. Bock^3^, Susanne Ohmann^1,2^, Petra Sackl-Pammer^1,2^, Sonja Werneck-Rohrer^1,2^, Harald Eder^1,2^, Katrin Skala^1,2^, Klara Czernin^1,2^, Dunja Mairhofer^1,2^, Bernhard Rohringer^1,2^, Carolin Bedus^1^, Ronja Lipp^1^, Christine Vesely^1,2^, Paul L. Plener^1,2,4^, Oswald D. Kothgassner^1,2^

Correspondence to: Mag. Dr. Oswald D. Kothgassner, Department of Child and Adolescent Psychiatry, Medical University of Vienna, E-Mail: [oswald.kothgassner@meduniwien.ac.at](mailto:oswald.kothgassner@meduniwien.ac.at)

**1: Model Assumptions of Our Confirmatory Analyses**

In this supplementary material, we report assumption tests of multiple regression analyses. The two conspiracy beliefs variables (i.e., specific and generic) were highly correlated in our sample (*r* = .86, *p* < .001). However, they were modeled in separate analyses as separate predictors, and multicollinearity was not an issue or a statistical assumption for these two conspiracy beliefs variables. All subsequent assumption tests of the remaining variables are reported below.

**Associations of Specific Conspiracy Beliefs and Adherence to Protective Guidelines**

For our first multiple regression, the data met the assumption of collinearity (all VIFs < 1.06), normality (Shapiro-Wilk *p* = .459), and independent errors (Durbin-Watson value = 2.45). Supplementary Figure 1 depicts additional model diagnostics—and visual inspection demonstrates that the data also met the assumptions of homogeneity of variance and linearity.

Figure S1: Visual check of model assumptions for the multiple regression of COVID-19-specific conspiracy beliefs and adherence to protective guidelines.


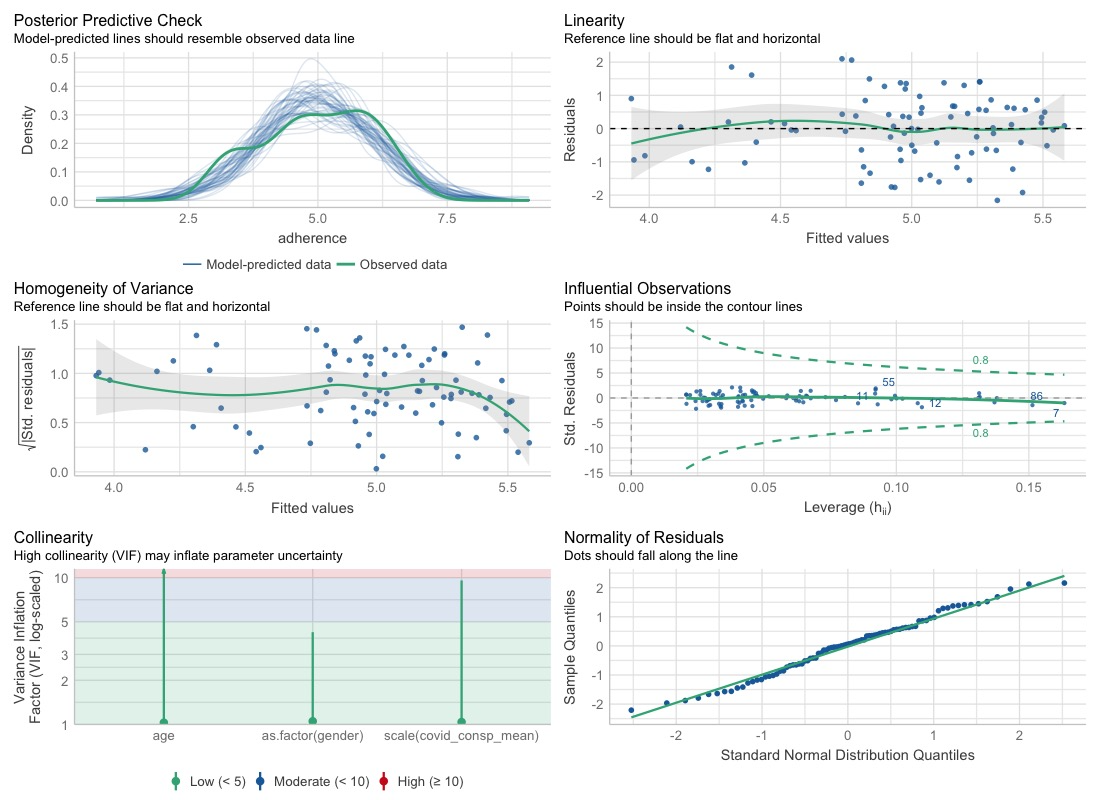


**Associations of Generic Conspiracy Beliefs and Adherence to Protective Guidelines**

For our second multiple regression, the data met the assumption of collinearity (all VIFs < 1.04), normality (Shapiro-Wilk *p* = .314), and independent errors (Durbin-Watson value = 2.48). Supplementary Figure 2 depicts additional model diagnostics—and visual inspection demonstrates that the data also met the assumptions of homogeneity of variance and linearity.

Figure S2: Visual check of model assumptions for the multiple regression of generic conspiracy beliefs and adherence to protective guidelines.


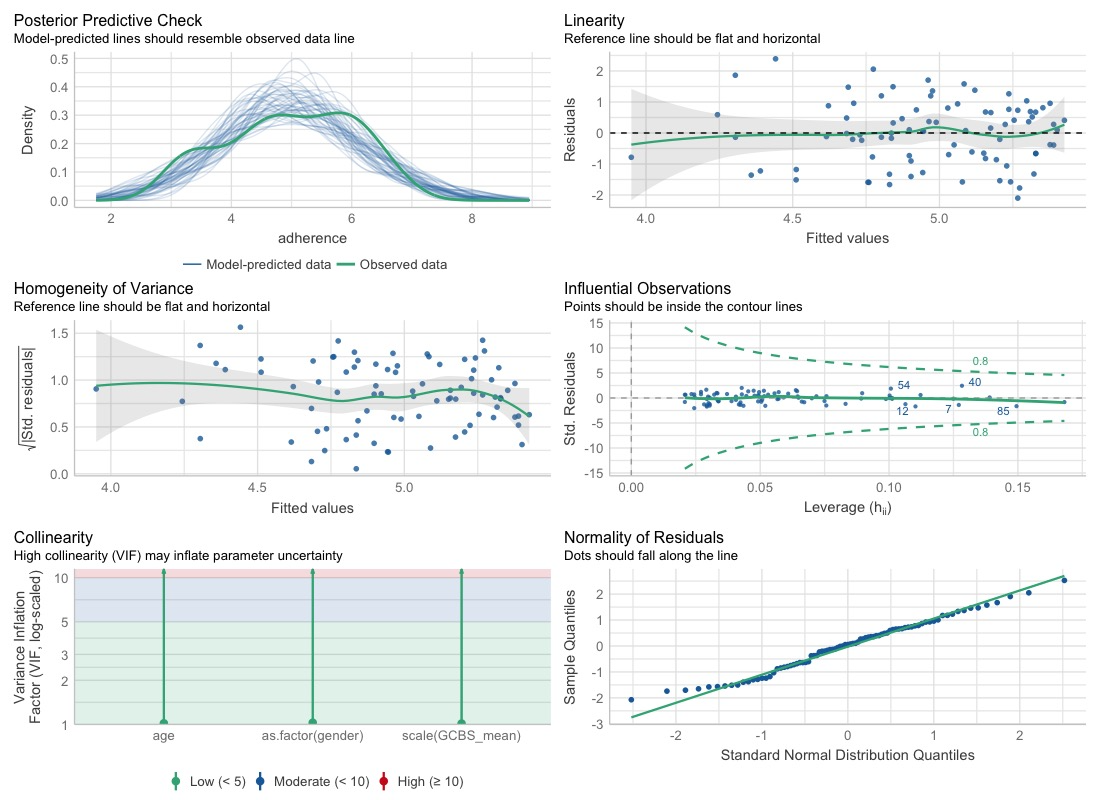

Supplement: Supplementary file 1 — Additional file 1. 1: Model assumptions of our confirmatory analyses. Figure S1: Visual check of model assumptions for the multiple regression of COVID-19-specific conspiracy beliefs and adherence to protective guidelines. Figure S2: Visual check of model assumptions for the multiple regression of generic conspiracy beliefs and adherence to protective guidelines. [file 13034_2022_554_MOESM1_ESM.docx]
